# Ethyl (2*S*,4*R*)-4-(4-bromo­phen­yl)-2-hydr­oxy-5,10-dioxo-3,4,5,10-tetra­hydro-2*H*-benzo[*g*]chromene-2-carboxyl­ate

**DOI:** 10.1107/S1600536810001315

**Published:** 2010-01-16

**Authors:** Wei Zhang, Yifeng Wang, Guangcun Zhang, Xiangsheng Xu

**Affiliations:** a State Key Laboratory Breeding Base of Green Chemistry–Synthesis Technology, Zhejiang University of Technology, Hangzhou 310014, People’s Republic of China

## Abstract

In the crystal structure of the title compound, C_22_H_17_BrO_6_, the quinone ring makes a dihedral angle of 81.84 (3)° with the benzene ring. The chiral C atoms, *viz.* the ring C atoms bearing the hydr­oxy and bromo­phenyl substituents, exhibit *R* and *S* configurations, respectively. The terminal ethyl group of the –CO_2_CH_2_CH_3_ group is disordered over two sets of sites with site-occupancy factors of 0.64 (1) and 0.36 (1). Inter­molecular O—H⋯O inter­actions further stabilize the crystal packing.

## Related literature

For general background to the modification of hydroxyquinone, see: Rueping *et al.* (2008 ▶); Zhou *et al.* (2008 ▶). For related structures, see: Peng (2006 ▶); Nasiri *et al.* (2008 ▶).
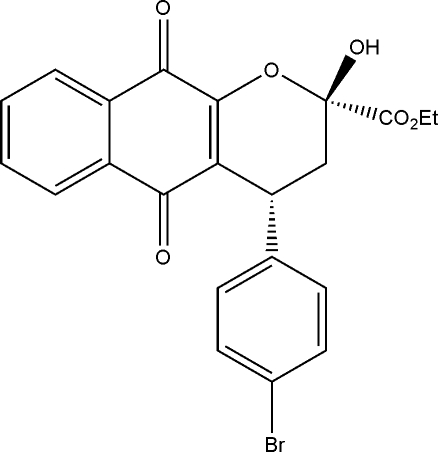


## Experimental

### 

#### Crystal data

C_22_H_17_BrO_6_
*M* *_r_* = 457.27
Monoclinic, 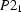

*a* = 8.2993 (6) Å
*b* = 9.9445 (7) Å
*c* = 12.4884 (10) Å
β = 96.323 (2)°
*V* = 1024.43 (13) Å^3^
*Z* = 2
Mo *K*α radiation
μ = 2.04 mm^−1^
*T* = 296 K
0.32 × 0.30 × 0.28 mm

#### Data collection

Rigaku R-AXIS RAPID diffractometer
Absorption correction: multi-scan (*ABSCOR*; Higashi, 1995 ▶) *T*_min_ = 0.520, *T*_max_ = 0.565
9887 measured reflections
4549 independent reflections
2257 reflections with *I* > 2σ(*I*)
*R*_int_ = 0.036

#### Refinement

*R*[*F* ^2^ > 2σ(*F* ^2^)] = 0.037
*wR*(*F* ^2^) = 0.122
*S* = 1.00
4549 reflections
273 parameters
3 restraints
H-atom parameters constrained
Δρ_max_ = 0.30 e Å^−3^
Δρ_min_ = −0.37 e Å^−3^
Absolute structure: Flack (1983 ▶), 2082 Friedel pairs
Flack parameter: −0.008 (11)

### 

Data collection: *PROCESS-AUTO* (Rigaku, 2006 ▶); cell refinement: *PROCESS-AUTO*; data reduction: *CrystalStructure* (Rigaku, 2007 ▶); program(s) used to solve structure: *SHELXS97* (Sheldrick, 2008 ▶); program(s) used to refine structure: *SHELXL97* (Sheldrick, 2008 ▶); molecular graphics: *ORTEP-3 for Windows* (Farrugia, 1997 ▶); software used to prepare material for publication: *WinGX* (Farrugia, 1999 ▶).

## Supplementary Material

Crystal structure: contains datablocks global, I. DOI: 10.1107/S1600536810001315/zq2026sup1.cif

Structure factors: contains datablocks I. DOI: 10.1107/S1600536810001315/zq2026Isup2.hkl

Additional supplementary materials:  crystallographic information; 3D view; checkCIF report

## Figures and Tables

**Table 1:** Hydrogen-bond geometry (Å, °)

*D*—H⋯*A*	*D*—H	H⋯*A*	*D*⋯*A*	*D*—H⋯*A*
O4—H4⋯O1^i^	0.82	1.90	2.716 (4)	177

Symmetry code: (i) 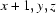.

**Fig. 1.**
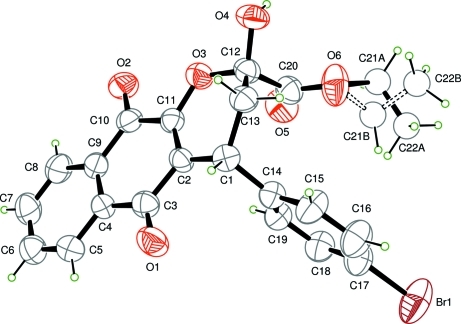
Molecular structure of the title compound, with the atomic labeling scheme. Displacement ellipsoids are drawn at the 50% probability level.

**Fig. 2.**
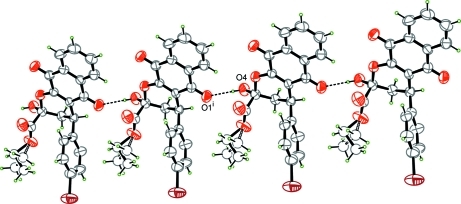
Crystal packing of the title compound showing O—H···O interactions (symmetry code i = x+1, y, z).

## supplementary crystallographic information

### Comment

The Michael addition to α, β-unsaturated systems is an important carbon-carbon bond-forming reaction in organic synthesis. Hydroxyquinones, quinones bearing a hydroxy group on the quinone ring, are an important class of the naturally occurring quinones with diverse biological activity. The title compound, ethyl (2*S*,4*R*)-4-(4-bromophenyl)-2-hydroxy-5,10-dioxo-3,4,5,10-tetrahydro-2*H*-benzo[*g*]chromene-2-carboxylate, was synthesized from a Michael Addition of 2-hydroxy-1,4-naphthoquinone to β,γ-unsaturated α-keto esters. The crystal structure of the title compound (Fig. 1) contains a tricyclic ring system with two chiral centers, which is consisting of a quinone ring and a tetrahydropyrane. One carbon atom of the tetrahydropyrane structure is not coplanar with the backbone, lying 0.554 (4) Å from the mean plane of the rest backbone. The terminal ethyl group of CO_2_CH_2_CH_3_ is disordered over two sites with site occupancy factors of 0.64 (1) and 0.36 (1). Moreover, weak O-H···O and C-H···O intermolecular interactions further stabilize the crystal structure.

### Experimental

To a solution of (*E*)-ethyl 4-(4-bromophenyl)-2-oxobut-3-enoate (1 mmol) and 2-hydroxy-1,4-naphthoquinone (1 mmol) in 1,4-dioxane (3 ml) was added 3-((1*S*)-(6-methoxyquinolin-4-yl) (8-vinylquinuclidin-2-yl)methylamino)-4- ((*S*)-1-phenylethylamino)cyclobut -3-ene-1,2-dione (0.025 mmol) as catalyst, and the mixture was stirred at room temperature for 12 h (monitored by TLC). Then the solvent was distilled under vacuum, and the residue was purified by flash column chromatography (silica gel, Hex/AcOEt, *v*/*v*, 3:1) giving the title compound. Single crystals were obtained by slow evaporation of an ethyl acetate solution.

### Refinement

H atoms were placed in calculated positions with C—H = 0.98 Å (*sp*), C—H = 0.97 (1) Å (sp^2^), C—H = 0.96 (1) Å (sp^3^), C—H = 0.93 (1) Å (aromatic) and *U*_iso_(H) = 1.2*U*_eq_ of the carrier carbon atoms. There is a positional disorder of the terminal ethyl group of CO_2_CH_2_CH_3_, the corresponding atoms C21 and C22 were split into two sites with refined site occupancy factors of 0.64 (1) and 0.36 (1).

### Crystal data

**Table:**

C_22_H_17_BrO_6_	*F*(000) = 464
*M**_r_* = 457.27	*D*_x_ = 1.482 Mg m^−^^3^
Monoclinic, *P*2_1_	Mo *K*α radiation, λ = 0.71073 Å
Hall symbol: P 2yb	Cell parameters from 6026 reflections
*a* = 8.2993 (6) Å	θ = 3.1–27.4°
*b* = 9.9445 (7) Å	µ = 2.04 mm^−^^1^
*c* = 12.4884 (10) Å	*T* = 296 K
β = 96.323 (2)°	Block, yellow
*V* = 1024.43 (13) Å^3^	0.32 × 0.30 × 0.28 mm
*Z* = 2	

### Data collection

**Table:**

Rigaku R-AXIS RAPID diffractometer	4549 independent reflections
Radiation source: rolling anode	2257 reflections with *I* > 2σ(*I*)
graphite	*R*_int_ = 0.036
Detector resolution: 10.00 pixels mm^-1^	θ_max_ = 27.4°, θ_min_ = 3.1°
ω scans	*h* = −10→9
Absorption correction: multi-scan (*ABSCOR*; Higashi, 1995)	*k* = −12→12
*T*_min_ = 0.520, *T*_max_ = 0.565	*l* = −16→16
9887 measured reflections	

### Refinement

**Table:**

Refinement on *F*^2^	Hydrogen site location: inferred from neighbouring sites
Least-squares matrix: full	H-atom parameters constrained
*R*[*F*^2^ > 2σ(*F*^2^)] = 0.037	*w* = 1/[σ^2^(*F*_o_^2^) + (0.0455*P*)^2^ + 0.125*P*] where *P* = (*F*_o_^2^ + 2*F*_c_^2^)/3
*wR*(*F*^2^) = 0.122	(Δ/σ)_max_ < 0.001
*S* = 1.00	Δρ_max_ = 0.30 e Å^−^^3^
4549 reflections	Δρ_min_ = −0.37 e Å^−^^3^
273 parameters	Extinction correction: *SHELXL97* (Sheldrick, 2008), Fc^*^=kFc[1+0.001xFc^2^λ^3^/sin(2θ)]^-1/4^
3 restraints	Extinction coefficient: 0.032 (3)
Primary atom site location: structure-invariant direct methods	Absolute structure: Flack (1983), 2082 Friedel pairs
Secondary atom site location: difference Fourier map	Flack parameter: −0.008 (11)

### Special details

**Table:**

Geometry. All e.s.d.'s (except the e.s.d. in the dihedral angle between two l.s. planes) are estimated using the full covariance matrix. The cell e.s.d.'s are taken into account individually in the estimation of e.s.d.'s in distances, angles and torsion angles; correlations between e.s.d.'s in cell parameters are only used when they are defined by crystal symmetry. An approximate (isotropic) treatment of cell e.s.d.'s is used for estimating e.s.d.'s involving l.s. planes.
Refinement. Refinement of *F*^2^ against ALL reflections. The weighted *R*-factor *wR* and goodness of fit *S* are based on *F*^2^, conventional *R*-factors *R* are based on *F*, with *F* set to zero for negative *F*^2^. The threshold expression of *F*^2^ > σ(*F*^2^) is used only for calculating *R*-factors(gt) *etc*. and is not relevant to the choice of reflections for refinement. *R*-factors based on *F*^2^ are statistically about twice as large as those based on *F*, and *R*- factors based on ALL data will be even larger.

### Fractional atomic coordinates and isotropic or equivalent isotropic displacement parameters (Å^2^)

**Table:**

	*x*	*y*	*z*	*U*_iso_*/*U*_eq_	Occ. (<1)
Br1	0.33160 (10)	0.08140 (7)	0.99547 (5)	0.1284 (4)	
O2	0.4425 (4)	0.3938 (3)	0.2686 (3)	0.0777 (8)	
O3	0.5431 (3)	0.4731 (3)	0.4648 (2)	0.0672 (7)	
O1	0.0040 (4)	0.4443 (3)	0.5476 (3)	0.0859 (10)	
O5	0.6613 (4)	0.2659 (3)	0.5810 (3)	0.0889 (10)	
C9	0.1656 (5)	0.3695 (4)	0.2984 (4)	0.0611 (10)	
O4	0.7341 (3)	0.5933 (3)	0.5658 (3)	0.0775 (8)	
H4	0.8167	0.5502	0.5593	0.093*	
C1	0.3205 (5)	0.5027 (4)	0.6295 (3)	0.0570 (10)	
H1	0.2390	0.5695	0.6443	0.068*	
O6	0.7358 (5)	0.3924 (4)	0.7236 (3)	0.1036 (12)	
C11	0.3827 (5)	0.4427 (4)	0.4456 (3)	0.0538 (10)	
C12	0.6071 (5)	0.5058 (4)	0.5745 (4)	0.0606 (10)	
C4	0.0525 (5)	0.3817 (4)	0.3718 (3)	0.0590 (10)	
C2	0.2738 (5)	0.4529 (4)	0.5170 (3)	0.0531 (9)	
C14	0.3196 (4)	0.3945 (4)	0.7159 (3)	0.0569 (10)	
C15	0.3232 (6)	0.4341 (4)	0.8218 (4)	0.0771 (13)	
H15	0.3219	0.5255	0.8375	0.093*	
C13	0.4825 (4)	0.5773 (5)	0.6336 (3)	0.0615 (9)	
H13A	0.4633	0.6660	0.6025	0.074*	
H13B	0.5261	0.5890	0.7083	0.074*	
C8	0.1163 (7)	0.3247 (5)	0.1949 (4)	0.0797 (13)	
H8	0.1922	0.3135	0.1461	0.096*	
C7	−0.0437 (7)	0.2971 (5)	0.1644 (4)	0.0874 (15)	
H7	−0.0758	0.2688	0.0943	0.105*	
C19	0.3191 (5)	0.2591 (4)	0.6952 (4)	0.0629 (11)	
H19	0.3151	0.2295	0.6243	0.076*	
C3	0.1041 (5)	0.4276 (4)	0.4823 (4)	0.0630 (11)	
C10	0.3394 (5)	0.4014 (4)	0.3315 (4)	0.0618 (11)	
C20	0.6695 (5)	0.3722 (5)	0.6254 (4)	0.0685 (11)	
C5	−0.1090 (5)	0.3531 (4)	0.3391 (4)	0.0707 (11)	
H5	−0.1852	0.3627	0.3879	0.085*	
C6	−0.1585 (6)	0.3107 (5)	0.2360 (4)	0.0795 (14)	
H6	−0.2670	0.2915	0.2148	0.095*	
C16	0.3287 (6)	0.3430 (5)	0.9056 (4)	0.0887 (14)	
H16	0.3334	0.3723	0.9766	0.106*	
C18	0.3245 (5)	0.1658 (4)	0.7774 (4)	0.0755 (13)	
H18	0.3264	0.0744	0.7616	0.091*	
C17	0.3270 (6)	0.2071 (5)	0.8817 (4)	0.0789 (13)	
C21A	0.8604 (17)	0.2834 (14)	0.7646 (12)	0.132 (5)	0.640 (10)
H21A	0.8698	0.2138	0.7112	0.159*	0.640 (10)
H21B	0.9665	0.3215	0.7868	0.159*	0.640 (10)
C22A	0.7845 (17)	0.2353 (15)	0.8537 (12)	0.147 (4)	0.640 (10)
H22A	0.8593	0.1800	0.8983	0.221*	0.640 (10)
H22B	0.6907	0.1833	0.8280	0.221*	0.640 (10)
H22C	0.7523	0.3101	0.8950	0.221*	0.640 (10)
C21B	0.763 (3)	0.2550 (18)	0.780 (3)	0.132 (5)	0.360 (10)
H21C	0.6731	0.2300	0.8194	0.159*	0.360 (10)
H21D	0.7829	0.1840	0.7297	0.159*	0.360 (10)
C22B	0.903 (3)	0.289 (3)	0.849 (2)	0.147 (4)	0.360 (10)
H22D	0.8842	0.2739	0.9224	0.221*	0.360 (10)
H22E	0.9294	0.3819	0.8392	0.221*	0.360 (10)
H22F	0.9924	0.2339	0.8322	0.221*	0.360 (10)

### Atomic displacement parameters (Å^2^)

**Table:**

	*U*^11^	*U*^22^	*U*^33^	*U*^12^	*U*^13^	*U*^23^
Br1	0.2086 (8)	0.0939 (4)	0.0809 (4)	0.0050 (5)	0.0071 (4)	0.0256 (3)
O2	0.0728 (19)	0.096 (2)	0.068 (2)	−0.0024 (17)	0.0246 (16)	−0.0008 (16)
O3	0.0554 (17)	0.0802 (18)	0.067 (2)	−0.0018 (14)	0.0092 (14)	0.0006 (15)
O1	0.0552 (17)	0.128 (3)	0.075 (2)	0.0008 (16)	0.0111 (17)	−0.0089 (19)
O5	0.094 (2)	0.0616 (19)	0.109 (3)	0.0086 (17)	0.005 (2)	−0.0157 (18)
C9	0.063 (3)	0.062 (2)	0.057 (3)	−0.002 (2)	0.002 (2)	0.0062 (19)
O4	0.0591 (16)	0.0631 (16)	0.110 (2)	−0.0151 (15)	0.0098 (17)	0.004 (2)
C1	0.060 (2)	0.051 (2)	0.061 (3)	0.0031 (18)	0.011 (2)	−0.0041 (18)
O6	0.132 (3)	0.079 (2)	0.091 (3)	0.025 (2)	−0.027 (2)	0.006 (2)
C11	0.054 (2)	0.052 (2)	0.056 (3)	0.0022 (16)	0.006 (2)	0.0052 (17)
C12	0.055 (2)	0.058 (2)	0.067 (3)	−0.0071 (19)	0.002 (2)	−0.0010 (19)
C4	0.051 (2)	0.062 (2)	0.062 (3)	−0.0003 (18)	0.000 (2)	0.0078 (19)
C2	0.042 (2)	0.055 (2)	0.062 (3)	0.0040 (16)	0.0023 (19)	0.0026 (17)
C14	0.062 (2)	0.053 (2)	0.055 (3)	−0.0003 (19)	0.0055 (19)	−0.0066 (18)
C15	0.118 (4)	0.053 (2)	0.062 (3)	−0.006 (2)	0.016 (3)	−0.004 (2)
C13	0.063 (2)	0.0481 (18)	0.073 (3)	−0.001 (2)	0.0057 (19)	0.001 (2)
C8	0.092 (4)	0.094 (3)	0.053 (3)	−0.003 (3)	0.009 (3)	0.001 (2)
C7	0.091 (4)	0.103 (4)	0.063 (3)	−0.008 (3)	−0.013 (3)	−0.005 (3)
C19	0.071 (3)	0.058 (2)	0.060 (3)	−0.0003 (19)	0.009 (2)	−0.0053 (19)
C3	0.060 (3)	0.064 (2)	0.066 (3)	0.0035 (18)	0.010 (2)	−0.0004 (19)
C10	0.063 (3)	0.057 (2)	0.066 (3)	−0.002 (2)	0.013 (2)	0.010 (2)
C20	0.062 (3)	0.065 (3)	0.077 (3)	0.004 (2)	0.002 (2)	0.000 (2)
C5	0.062 (3)	0.077 (3)	0.073 (3)	−0.006 (2)	0.007 (2)	0.002 (2)
C6	0.077 (3)	0.082 (3)	0.076 (4)	−0.007 (2)	−0.006 (3)	0.003 (3)
C16	0.126 (4)	0.085 (3)	0.056 (3)	0.002 (3)	0.014 (3)	−0.012 (2)
C18	0.093 (4)	0.057 (3)	0.075 (3)	−0.003 (2)	0.005 (3)	−0.001 (2)
C17	0.097 (4)	0.073 (3)	0.065 (3)	0.004 (2)	0.004 (3)	−0.005 (2)
C21A	0.160 (14)	0.136 (8)	0.092 (7)	−0.003 (9)	−0.021 (10)	0.026 (7)
C22A	0.133 (10)	0.180 (12)	0.129 (9)	−0.002 (9)	0.013 (9)	0.027 (9)
C21B	0.160 (14)	0.136 (8)	0.092 (7)	−0.003 (9)	−0.021 (10)	0.026 (7)
C22B	0.133 (10)	0.180 (12)	0.129 (9)	−0.002 (9)	0.013 (9)	0.027 (9)

### Geometric parameters (Å, °)

**Table:**

Br1—C17	1.889 (5)	C15—H15	0.9300
O2—C10	1.226 (5)	C13—H13A	0.9700
O3—C11	1.360 (5)	C13—H13B	0.9700
O3—C12	1.451 (5)	C8—C7	1.368 (7)
O1—C3	1.238 (5)	C8—H8	0.9300
O5—C20	1.192 (5)	C7—C6	1.384 (7)
C9—C8	1.386 (6)	C7—H7	0.9300
C9—C4	1.387 (6)	C19—C18	1.381 (6)
C9—C10	1.490 (6)	C19—H19	0.9300
O4—C12	1.380 (5)	C5—C6	1.374 (7)
O4—H4	0.8200	C5—H5	0.9300
C1—C2	1.500 (5)	C6—H6	0.9300
C1—C14	1.524 (6)	C16—C17	1.383 (6)
C1—C13	1.531 (5)	C16—H16	0.9300
C1—H1	0.9800	C18—C17	1.364 (6)
O6—C20	1.304 (5)	C18—H18	0.9300
O6—C21B	1.541 (12)	C21A—C22A	1.420 (13)
O6—C21A	1.546 (11)	C21A—H21A	0.9700
C11—C2	1.341 (6)	C21A—H21B	0.9700
C11—C10	1.489 (6)	C22A—H22A	0.9600
C12—C13	1.513 (6)	C22A—H22B	0.9600
C12—C20	1.538 (6)	C22A—H22C	0.9600
C4—C5	1.387 (6)	C21B—C22B	1.415 (14)
C4—C3	1.472 (6)	C21B—H21C	0.9700
C2—C3	1.450 (6)	C21B—H21D	0.9700
C14—C19	1.371 (5)	C22B—H22D	0.9600
C14—C15	1.377 (6)	C22B—H22E	0.9600
C15—C16	1.381 (6)	C22B—H22F	0.9600
			
C11—O3—C12	117.8 (3)	C14—C19—C18	121.4 (4)
C8—C9—C4	119.5 (4)	C14—C19—H19	119.3
C8—C9—C10	120.3 (4)	C18—C19—H19	119.3
C4—C9—C10	120.2 (4)	O1—C3—C2	118.7 (4)
C12—O4—H4	109.5	O1—C3—C4	120.9 (4)
C2—C1—C14	114.1 (3)	C2—C3—C4	120.5 (4)
C2—C1—C13	109.0 (3)	O2—C10—C11	121.2 (4)
C14—C1—C13	113.0 (3)	O2—C10—C9	122.1 (4)
C2—C1—H1	106.7	C11—C10—C9	116.6 (4)
C14—C1—H1	106.7	O5—C20—O6	124.8 (4)
C13—C1—H1	106.7	O5—C20—C12	125.1 (4)
C20—O6—C21B	108.5 (11)	O6—C20—C12	110.1 (4)
C20—O6—C21A	113.7 (7)	C6—C5—C4	121.2 (4)
C2—C11—O3	125.7 (4)	C6—C5—H5	119.4
C2—C11—C10	123.2 (4)	C4—C5—H5	119.4
O3—C11—C10	111.0 (3)	C5—C6—C7	118.7 (5)
O4—C12—O3	105.7 (3)	C5—C6—H6	120.7
O4—C12—C13	108.1 (3)	C7—C6—H6	120.7
O3—C12—C13	111.6 (3)	C15—C16—C17	118.6 (4)
O4—C12—C20	110.6 (3)	C15—C16—H16	120.7
O3—C12—C20	105.5 (3)	C17—C16—H16	120.7
C13—C12—C20	114.9 (4)	C17—C18—C19	120.2 (4)
C9—C4—C5	119.4 (4)	C17—C18—H18	119.9
C9—C4—C3	119.9 (4)	C19—C18—H18	119.9
C5—C4—C3	120.8 (4)	C18—C17—C16	119.9 (4)
C11—C2—C3	119.4 (4)	C18—C17—Br1	121.0 (4)
C11—C2—C1	121.6 (3)	C16—C17—Br1	119.0 (4)
C3—C2—C1	118.7 (4)	C22A—C21A—O6	99.0 (10)
C19—C14—C15	117.5 (4)	C22A—C21A—H21A	112.0
C19—C14—C1	124.0 (4)	O6—C21A—H21A	112.0
C15—C14—C1	118.5 (3)	C22A—C21A—H21B	112.0
C14—C15—C16	122.4 (4)	O6—C21A—H21B	112.0
C14—C15—H15	118.8	H21A—C21A—H21B	109.6
C16—C15—H15	118.8	C22B—C21B—O6	97.8 (16)
C12—C13—C1	113.6 (3)	C22B—C21B—H21C	112.2
C12—C13—H13A	108.8	O6—C21B—H21C	112.2
C1—C13—H13A	108.8	C22B—C21B—H21D	112.2
C12—C13—H13B	108.8	O6—C21B—H21D	112.2
C1—C13—H13B	108.8	H21C—C21B—H21D	109.8
H13A—C13—H13B	107.7	C21B—C22B—H22D	109.5
C7—C8—C9	120.1 (5)	C21B—C22B—H22E	109.5
C7—C8—H8	120.0	H22D—C22B—H22E	109.5
C9—C8—H8	120.0	C21B—C22B—H22F	109.5
C8—C7—C6	121.1 (5)	H22D—C22B—H22F	109.5
C8—C7—H7	119.4	H22E—C22B—H22F	109.5
C6—C7—H7	119.4		
			
C12—O3—C11—C2	7.0 (5)	C9—C4—C3—O1	177.1 (4)
C12—O3—C11—C10	−175.6 (3)	C5—C4—C3—O1	−1.6 (6)
C11—O3—C12—O4	−150.5 (3)	C9—C4—C3—C2	−3.5 (5)
C11—O3—C12—C13	−33.2 (4)	C5—C4—C3—C2	177.9 (4)
C11—O3—C12—C20	92.3 (4)	C2—C11—C10—O2	179.7 (4)
C8—C9—C4—C5	−2.1 (6)	O3—C11—C10—O2	2.2 (5)
C10—C9—C4—C5	179.3 (4)	C2—C11—C10—C9	−0.5 (5)
C8—C9—C4—C3	179.2 (4)	O3—C11—C10—C9	−178.1 (3)
C10—C9—C4—C3	0.6 (5)	C8—C9—C10—O2	2.5 (6)
O3—C11—C2—C3	174.9 (3)	C4—C9—C10—O2	−178.8 (4)
C10—C11—C2—C3	−2.3 (5)	C8—C9—C10—C11	−177.3 (4)
O3—C11—C2—C1	0.8 (6)	C4—C9—C10—C11	1.4 (5)
C10—C11—C2—C1	−176.3 (3)	C21B—O6—C20—O5	−13.4 (15)
C14—C1—C2—C11	−109.5 (4)	C21A—O6—C20—O5	22.5 (9)
C13—C1—C2—C11	18.0 (5)	C21B—O6—C20—C12	167.8 (14)
C14—C1—C2—C3	76.5 (4)	C21A—O6—C20—C12	−156.2 (7)
C13—C1—C2—C3	−156.1 (3)	O4—C12—C20—O5	−115.1 (5)
C2—C1—C14—C19	16.4 (5)	O3—C12—C20—O5	−1.3 (5)
C13—C1—C14—C19	−108.9 (4)	C13—C12—C20—O5	122.1 (5)
C2—C1—C14—C15	−165.1 (4)	O4—C12—C20—O6	63.6 (5)
C13—C1—C14—C15	69.5 (5)	O3—C12—C20—O6	177.5 (4)
C19—C14—C15—C16	1.0 (7)	C13—C12—C20—O6	−59.2 (5)
C1—C14—C15—C16	−177.6 (4)	C9—C4—C5—C6	1.1 (6)
O4—C12—C13—C1	168.5 (3)	C3—C4—C5—C6	179.8 (4)
O3—C12—C13—C1	52.7 (4)	C4—C5—C6—C7	−0.2 (7)
C20—C12—C13—C1	−67.4 (4)	C8—C7—C6—C5	0.2 (8)
C2—C1—C13—C12	−44.0 (4)	C14—C15—C16—C17	−1.4 (8)
C14—C1—C13—C12	84.1 (4)	C14—C19—C18—C17	1.4 (7)
C4—C9—C8—C7	2.1 (7)	C19—C18—C17—C16	−1.8 (7)
C10—C9—C8—C7	−179.2 (4)	C19—C18—C17—Br1	179.2 (3)
C9—C8—C7—C6	−1.2 (8)	C15—C16—C17—C18	1.8 (8)
C15—C14—C19—C18	−0.9 (6)	C15—C16—C17—Br1	−179.2 (4)
C1—C14—C19—C18	177.5 (4)	C20—O6—C21A—C22A	−119.4 (11)
C11—C2—C3—O1	−176.2 (4)	C21B—O6—C21A—C22A	−31 (2)
C1—C2—C3—O1	−2.0 (5)	C20—O6—C21B—C22B	149 (2)
C11—C2—C3—C4	4.3 (5)	C21A—O6—C21B—C22B	43.5 (16)
C1—C2—C3—C4	178.5 (3)		

### Hydrogen-bond geometry (Å, °)

**Table:**

*D*—H···*A*	*D*—H	H···*A*	*D*···*A*	*D*—H···*A*
O4—H4···O1^i^	0.82	1.90	2.716 (4)	177

Symmetry codes: (i) *x*+1, *y*, *z*.
